# Identification of microbial pathogens in Neolithic Scandinavian humans

**DOI:** 10.1038/s41598-024-56096-0

**Published:** 2024-03-07

**Authors:** Nora Bergfeldt, Emrah Kırdök, Nikolay Oskolkov, Claudio Mirabello, Per Unneberg, Helena Malmström, Magdalena Fraser, Federico Sanchez-Quinto, Roger Jorgensen, Birgitte Skar, Kerstin Lidén, Mattias Jakobsson, Jan Storå, Anders Götherström

**Affiliations:** 1 Centre for Palaeogenetics, Stockholm University, Stockholm, Sweden; 2 Department of Zoology, Stockholm University, Stockholm, Sweden; 3 Department of Bioinformatics and Genetics, Swedish Museum of Natural History, Stockholm, Sweden; 4 Department of Biotechnology, Faculty of Science, Mersin University, Mersin, Turkey; 5 Science for Life Laboratory, Department of Biology, National Bioinformatics Infrastructure Sweden, Lund University, Lund, Sweden; 6 Science for Life Laboratory, Department of Physics, Chemistry and Biology, National Bioinformatics Infrastructure Sweden, Linköping University, Linköping, Sweden; 7 Science for Life Laboratory, Department of Cell and Molecular Biology, National Bioinformatics Infrastructure Sweden, Uppsala University, Uppsala, Sweden; 8 Human Evolution, Department of Organism Biology, Uppsala University, Uppsala, Sweden; 9 Tromsø University Museum, University of Tromsø-The Arctic University of Norway, Tromsø, Norway; 10 Department of Archaeology and Cultural History, NTNU University Museum, Trondheim, Norway; 11 Department of Archaeology and Classical Studies, Stockholm University, Stockholm, Sweden

**Keywords:** Archaeology, Microbial genetics

## Abstract

With the Neolithic transition, human lifestyle shifted from hunting and gathering to farming. This change altered subsistence patterns, cultural expression, and population structures as shown by the archaeological/zooarchaeological record, as well as by stable isotope and ancient DNA data. Here, we used metagenomic data to analyse if the transitions also impacted the microbiome composition in 25 Mesolithic and Neolithic hunter-gatherers and 13 Neolithic farmers from several Scandinavian Stone Age cultural contexts. *Salmonella enterica,* a bacterium that may have been the cause of death for the infected individuals, was found in two Neolithic samples from Battle Axe culture contexts. Several species of the bacterial genus *Yersinia* were found in Neolithic individuals from Funnel Beaker culture contexts as well as from later Neolithic context. Transmission of e.g. *Y. enterocolitica* may have been facilitated by the denser populations in agricultural contexts.

## Introduction

During the Neolithic transition, the human lifestyle and subsistence shifted from hunting and gathering to an agricultural lifestyle. In Europe, the transition appears to have been largely associated with mobility, seen in many recent archaeogenetic studies as a marked process of geneflow from Anatolia^1,2^. In Scandinavia, the Neolithic period started with the appearance of the cultural complex called the Funnel Beaker culture (FBC) c. 6000 years before present (BP)^3,4^. The FBC cultural complex, often with a major ancestry component deriving from Anatolian Neolithic farmers^1,5–7^, persisted until c. 4800 BP and was succeeded by the Battle Axe culture (BAC, c. 4800–4300 BP)^8^. However, an important hunter-gatherer cultural complex, the Pitted Ware culture (PWC), existed in parallel (c. 5400–4400 BP) with the above agricultural complexes, mainly along the coast of southern Scandinavia^9^. Individuals from a PWC context have been shown to share a large portion of genetic ancestry with chronologically older European Mesolithic hunter-gatherers^10^, and although Neolithic farmer and hunter-gatherer groups were genetically distinct, gene flow from PWC to FBC individuals has been detected^5^. Individuals from a BAC context seem to share most genetic ancestry with other groups on the European continent, especially the Corded Ware culture (CWC)^11^. However, while there is ample archaeological evidence that there were both social and cultural interactions between individuals from the PWC and BAC complexes in Scandinavia, so far there is no genetic evidence for interactions between the groups, as seen e.g. on Gotland^12^.

Apart from the change in subsistence strategies, the Neolithic transition entailed changes in demography^13^. Larger and more dense populations could potentially have provided environments where diseases could spread more easily^14^, increasing both the pathogen load and dispersal modes. Besides a higher population density, the migration of human populations itself, during this time, could also have facilitated the dispersal of human microbiomes. Further, it has been suggested that keeping domesticated livestock can promote zoonotic transmissions of diseases^15^.

Dispersal of microbes can now be approached using archaeogenetic methods and may provide important insights into living conditions and health in the past. By the introduction of high-throughput sequencing, specific diseases such as plague and paratyphoid fever have been possible to study in prehistoric/historic populations^16–19^. Moreover, metagenomic studies have made it possible to detect changes in the human microbiota over time and how these changes affected human prehistoric populations. For example, early Mesolithic hunter-gatherers in present-day Poland exhibited fewer caries- and periodontal disease-associated microbe taxa than Neolithic farming groups in present-day Germany^20^. This is in accordance with observations on the incidence of actual lesions in the dental record of hunter-gatherer and farmer groups. Further, studies based on coprolites on more modern human populations, industrialized versus non-industrialized, have shown differences in gut microbiome^21^.

Here, we performed a metagenomic analysis of aDNA extracted from teeth of 38 Stone Age individuals recovered from eleven sites in Scandinavia. The samples came from different cultural contexts: Mesolithic hunter-gatherers, named here (SHG); Neolithic hunter-gatherers from the Pitted Ware culture (PWC); two Neolithic cultures (FBC and BAC); and two chronologically younger samples from Late Neolithic Farmers (Ans004 and Ans010) from a dolmen^22^, named here (LNF). A sample from Steigen in Norway has been dated to the Neolithic period, but genetic data and isotope analysis indicates close affinity to Mesolithic hunter-gatherers^6^ and the sample is thus treated as a Mesolithic hunter-gatherer in this study. Our purpose was to investigate the microbial content of these skeletal samples to gain understanding of their health and living conditions. We also explored whether we could find microbes in more than one cultural context, which could indicate possible transmissions and thus contacts between Neolithic hunter-gatherer (PWC) and other Neolithic populations (FBC).

## Samples

**Figure 1:**
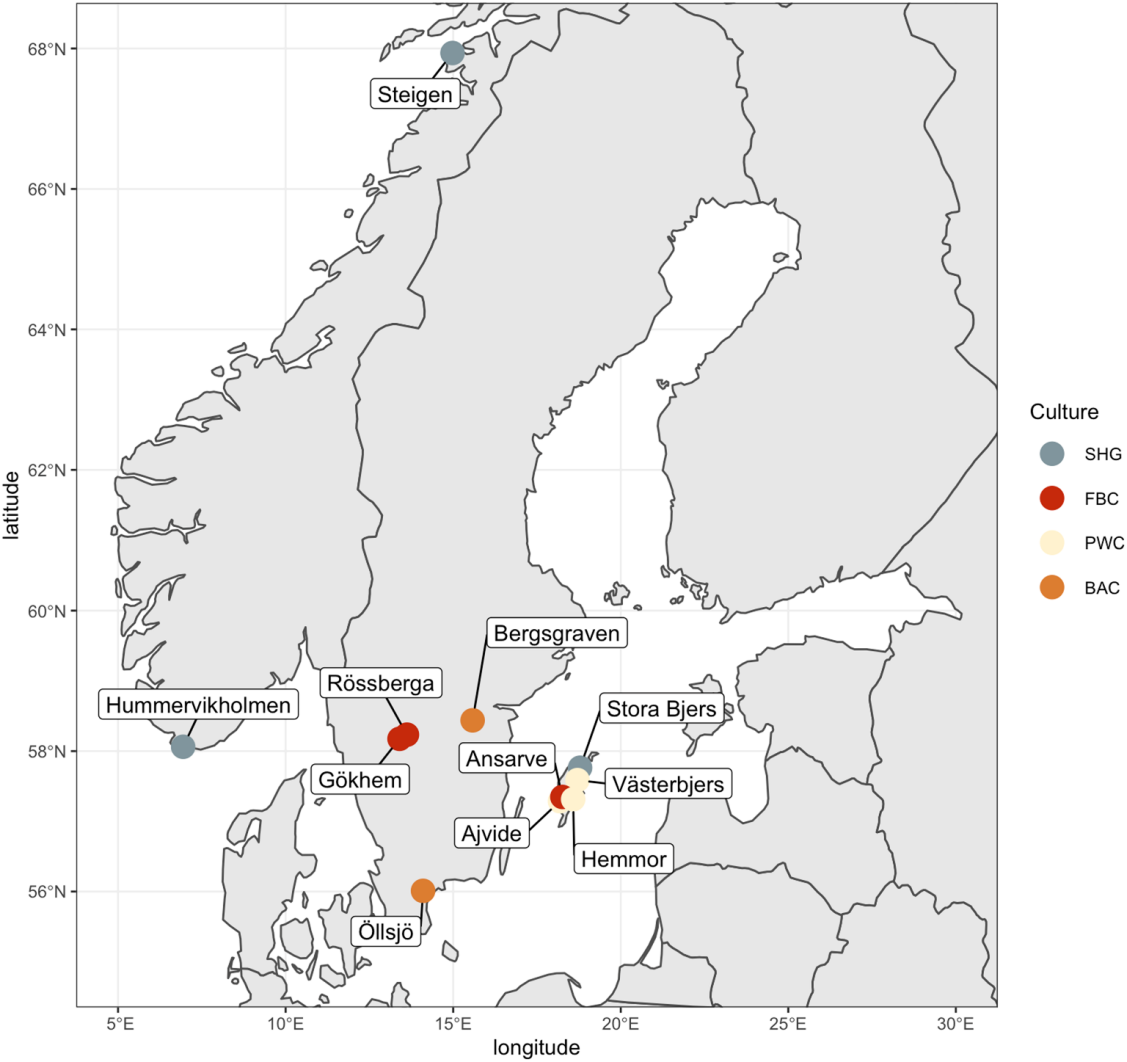
Map of Scandinavia showing the locations where samples were collected. SHG = Mesolithic Scandinavian hunter-gatherers, FBC = Funnel Beaker Culture, PWC = Pitted Ware Culture, BAC = Battle Axe Culture. The samples belonging to Late Neolithic Farmers (LNF) were collected from the FBC site Ansarve.

We analyzed shotgun-sequenced DNA from 38 individuals for microbial species. The samples came from eleven sites in Sweden and Norway, with different chronologies and different cultural affinities (Table 1, Fig. 1). Human aDNA from these individuals was previously investigated for 36 individuals, while two were uniquely sequenced for this study. Since bacterial DNA seems to be better preserved in teeth than in other skeletal parts^23^, we only used data from teeth in this study.

**Table 1:** List of samples included in this study.

Context	No of samples	Locations	References
Mesolithic hunter-gatherers (SHG)	4	Hummervikholmen, Stora Bjers, Steigen	^6^
Funnel beaker culture (FBC)	8	Ansarve, Gökhem, Rössberga	^1,5–7,11,22^, this study
Pitted ware culture (PWC)	21	Ajvide, Hemmor, Västerbjers	^1,5,6,11,12^, this study
Battle axe culture (BAC)	3	Bergsgraven, Öllsjö	^11^
Late neolithic farmers (LNF)	2	Ansarve	^22^

## Methods

The DNA for 36 of the individuals had been extracted and sequenced in previous studies (see Table S1), using demineralization and a protein denaturing agent, and purified using silica binding protocols. The two individuals that were not previously analysed (ajv049 and ajv082) were extracted following a silica-based protocol and sequenced on an Illumina HiSeq 2500 at SciLifeLab in Uppsala, all according to the procedure described in Günther et al.^6^.

After trimming adapters, using cutadapt^24^, and removing reads mapping to the human genome, we used the metagenomics pipeline aMeta^25^, which combines *k*-mer based taxonomic classification and alignment approaches for a fast and accurate identification of ancient microbial species. An initial *k*-mer based taxonomic classification using the KrakenUniq tool^26^ was followed by an alignment analysis with the Lowest Common Ancestor (LCA) algorithm, implemented in the MALT software^27^. For taxonomic profiling with KrakenUniq, we built an extended NCBI non-redundant (NT) database, consisting of microbial genomes (bacteria, viruses, archaea, fungi, and parasitic worms) as well as human genome and complete eukaryotic genomes available at NCBI. To validate and authenticate microorganisms detected by KrakenUniq, alignment analysis using MALT was performed using a custom reference database comprising 660 microbial genomes from the species identified by KrakenUniq.

To further authenticate the microbial organisms found in the human aDNA samples, we applied the MaltExtract tool^28^ to the LCA verified alignments produced by MALT, and computed deamination pattern, read length distribution, and edit distance authentication metrics. Additionally, breadth/evenness of coverage of reads aligned to each microbial organism was assessed using Samtools^29^. Finally, histograms of post-mortem damage (PMD) scores were computed using PMDtools^30^ to examine the ancient status of individual metagenomic reads.

To evaluate the ancient status of the bacterial species, we used eight different authentication metrics, further presented in the aMeta workflow^25^. Firstly, deamination patterns must be observed in the 5′ and 3′ ends of the ancient DNA reads. Moreover, if a microbe is truly present in a sample most of its reads align with 0, but still a fair amount with 1 mismatch which is quantified by the edit distance metric. To demonstrate the fragmentation of the aDNA reads, the read length distribution is evaluated. Further, reads could align to the conserved regions among the bacterial genomes and could create false positive assignments. To eliminate these false positives, the read distribution across the reference genome is assessed. These authentication metrics are then weighted against each other, and heavier weight is assigned to evenness of coverage and deamination profile than to the other metrics. The authentication scores are then presented in a heatmap summary where a higher value indicates an organism truly being present and ancient.

Further, microbial abundance quantification from rma6 MALT alignments was performed using MaltExtract and rma2info wrapper script from MEGAN tool^31^, as well as a custom awk script applied to sam-alignments from MALT.

### Ethical approval

Permission for using legal authority to collect and study teeth of the Stone Age individuals in the study was issued by Statens Historiska Museer (SHM). Permission to sample and perform the analyses of Ajvide-finds were obtained by Göran Burenhult and Inger Österholm, Gotland University College.

## Results and discussion

The KrakenUniq screening identified 660 microbial species in the metagenomic dataset of the 38 samples (Table S2). After performing MALT and the authentication steps, only DNA belonging to bacteria could be confirmed, while no fungal or viral organisms were authenticated. In other studies, viral DNA has been identified in human teeth dated to c. 31600 BP^32^, and fungal DNA has been detected in ancient samples^33^, although species assessment has not been possible. We therefore conclude, that despite that we cannot reject potential presence of viruses and fungi in our samples, these organisms did not have detectable levels of DNA fragments and thus could not be reliably authenticated.

**Figure 2:**
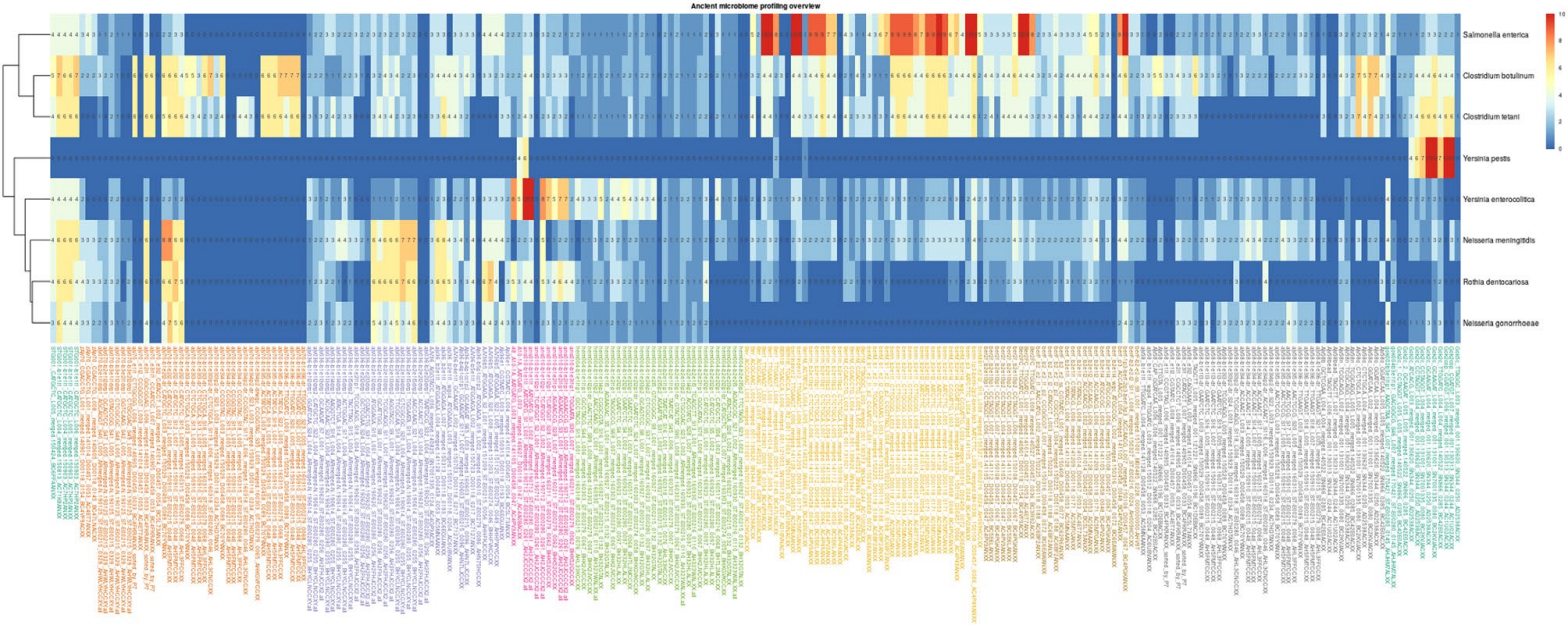
Heatmap summary of authentication scores of detected pathogens for individuals gok2c, ajv58, ber1, ber2, hem004, ans010, ajv36, ajv70, stg001.

In the dataset we found some potentially pathogenic bacterial species. In the hunter-gatherers (SHG), the initial KrakenUniq screening found sequences mapping to *Neisseria meningitidis*, known to cause meningococcal disease, in one individual (stg001), from Steigen in Norway dated to c. 5850 cal BP (Figs. 2, S1). This infection is commonly transmitted via saliva, for example through coughing, sneezing, and kissing. Common symptoms of meningitis include headache and fever, and though it is easily treated with modern healthcare, the infection can be fatal. *N. meningitidis* has previously been identified in human samples from Germany dated to c. 1000–750 BP^34^, as well as from California, North America, dated to 700–310 BP^35^. However, according to the authentication score of 6 (Fig. 2), it is not possible to unambiguously draw the conclusion of this species being present in the sample. With more data, the presence or absence of this organism should be possible to verify.

Indications of the presence of *N. meningitidis* were also found in two Middle Neolithic hunter-gatherer (PWC) individuals from Ajvide (ajv36 and ajv70), dated to c. 4800 and 4750 cal BP respectively (Figs. 2, S2–S3). The authentication scores for these individuals rank higher than that of stg001 with a score of 8 and 7 respectively (Fig. 2), however especially ajv36 presents an ambiguous picture. Given that it is the edit distance analyses that lowers the authentication score, it is possible that the result is obscured by a cohort of mismapped fragments, but it could also be a more variable strain that has gone extinct or has not yet been identified. We also found indications of another bacterium of the same genus: *N. gonorrhoeae*, which causes the sexually transmitted infection gonorrhoea. Gonorrhoea can lead to sepsis, which is potentially fatal, but more commonly it can lead to infertility and has also been connected to several foetal conditions. Previously, *N. gonorrhoeae* has been identified in samples from Germany dated to c. 1000–750 BP^34^. The indications are however ambiguous, and the presence of *N. gonorrhoeae* cannot be confirmed with certainty. We also found similar ambiguous indications of the presence of *Rothia dentocariosa* in these PWC individuals, a bacterium that can cause infections, but is primarily known to be a part of the healthy human oral microbiome (Figs. 2, S4).

In one of the FBC individuals, gok2, an adult female, we found the plague causing bacterium *Yersinia pestis* (Figs. 2, S5). This finding confirms the previously published results for this individual^19^, which is one of the earliest finding of *Y. pestis* to date. *Y. pestis* is transmitted to humans from infected rodents via flea bites. However, the mutation in the *ymt* gene that allows flea transmission of *Y. pestis* was not acquired until some hundred years later^36^, and the transmissibility in the population was thus likely low.

**Figure 3:**
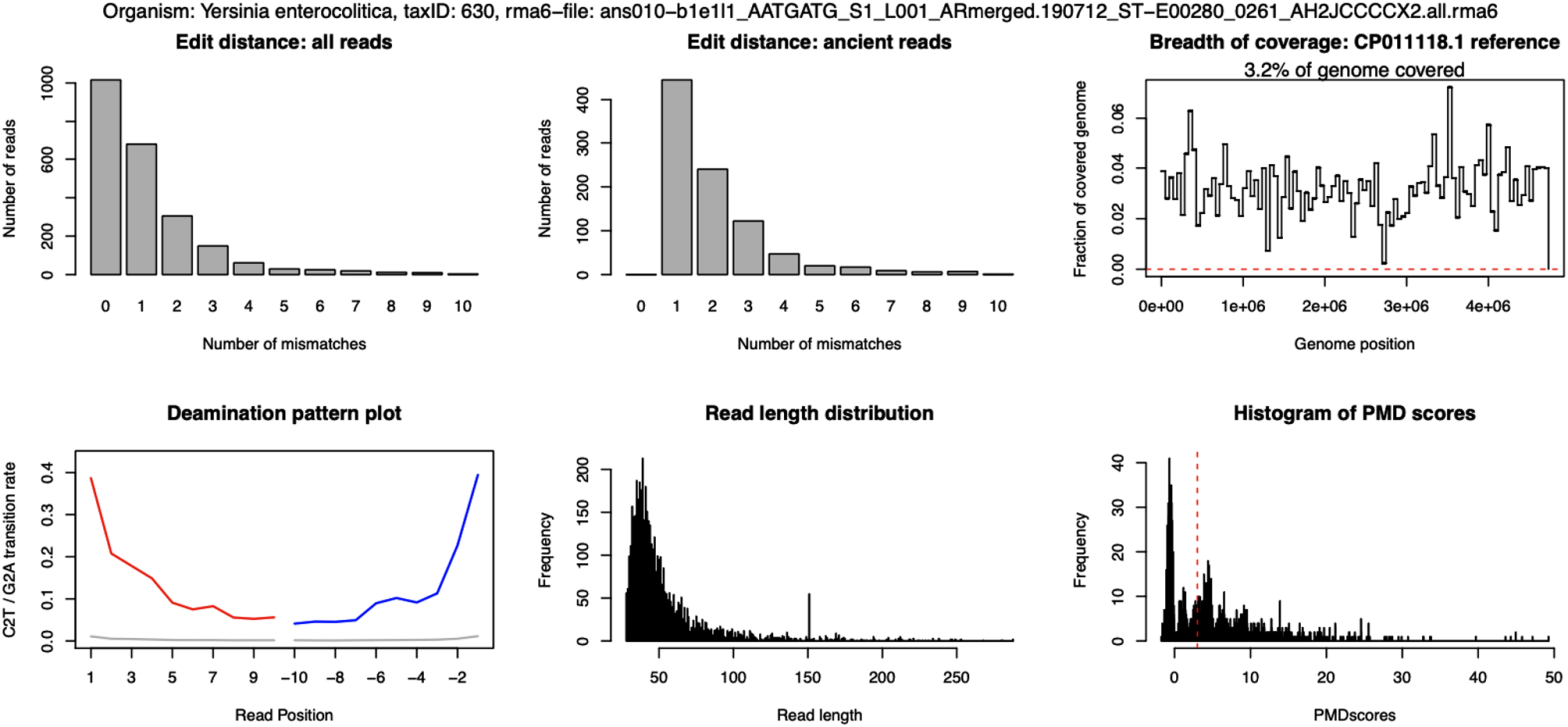
Edit distance, breadth of coverage, deamination plot, read length distribution of *Yersinia enterocolitica* in individual ans010.

A second species within the genus *Yersinia*, *Y. enterocolitica*, was found in a Late Neolithic individual (ans010, c. 3900 cal BP) buried in the Middle Neolithic FBC dolmen in Ansarve on Gotland (Figs. 2; 3). *Y. enterocolitica* causes yersiniosis, a possibly lethal infection with symptoms including diarrhoea and fever. The bacterium is typically spread from contaminated water and food, but there are also signs of some transmission between humans as well as zoonotic transmission^37^. In the initial screening, *Y. enterocolitica* was also detected in an individual from the Middle Neolithic hunter-gatherer PWC culture context from Hemmor, Gotland (hem004, c. 5000 cal BP). However, this could not be confirmed in later authentication steps due to the deamination patterns being irregular (Figs. 2, S6).

Several possibly pathogenic species were identified in the Neolithic contexts. In two individuals from a BAC context (ber001 and ber002), we found *Salmonella enterica*, the causing agent of salmonellosis (Figs. 2, S7–S8). *S. enterica* has previously been identified in samples from the Neolithic period, and the Neolithization, especially domestication of livestock, has likely played a big part in the evolution of the pathogens host-adaptivity^38^. The symptoms of salmonellosis include stomach cramps, fever, and diarrhoea, however, it is worth noting that there are 2600 serovars of *S. enterica*, divided into typhoidal an non-typhoidal, with differing epidemiology and clinical response^39^. In modern populations, *S. enterica* is typically transmitted to humans through ingestion of contaminated meat, eggs, or milk, though zoonotic transmission is also possible. Interestingly, these two individuals were recovered from the same grave^11^ and this finding could thus imply the cause of death. A third individual, an infant, was discovered after the initial excavation^11^, and future studies could investigate whether *S. enterica* is present also in these remains.

Some species of ancient bacteria were found across all investigated contexts. Among these were *Clostridium botulinum*, a bacterium commonly found in decaying flesh (Figs. 2, S9). We also found *Clostridium tetani* (Figs. 2, S10)*,* which can cause severe and potentially lethal infections in humans. However, both species are common in soil, and may thus have uncertain association to the individuals in which they were found. The authentication score presented in Fig. 2 gives further indications that these species may be present but are likely not ancient.

It is important to stress that these results are not a complete and comprehensive pathogenic diagnosis of the investigated remains. Although the stringent authentication procedure makes false positives unlikely, factors such as preservation, proportion of the DNA extract, and the location of different infections in the body make false negatives likely. This implies that we presumably do not discover all the variety of potential pathogenic factors due to the lack of material and data.

Ancient microbes detected in prehistoric oral material is now emerging as an important complement to other types of information on historic and prehistoric health^40^. In this study, we identified several possibly pathogenic bacteria in prehistoric samples that can help shed light, not only on the evolution of the bacteria, but also on the palaeopathology of past human groups. Notable is the finding of *S. enterica*, which likely influenced the general health of the Neolithic farmers that hosted the bacteria and is strongly associated to an agricultural lifestyle. We have further presented what is possibly some of the earliest pathogen findings of *N. meningitidis* in prehistoric humans so far. However, we acknowledge that the *Neisseria* sequences present an ambiguous case that likely can be resolved with further sequencing from these individuals. Presently, ajv70 should be viewed as a probable case of *N. meningitidis*. Moreover, we may have identified the cause-of-death for some of the investigated individuals, e.g. salmonellosis or yersinosis, something that is often extremely challenging if no visible trauma is present. Further, in the Neolithic contexts we encountered pathogens, such as *Y. pestis* and *Y. enterocolitica*, indicating more and frequent contact between humans and other humans as well as animals.

### Supplementary Information

Supplementary Information.

## Data Availability

Data will be made available in the European Nucleotide Archive, accession number PRJEB62680, https://www.ebi.ac.uk/ena/browser/view/PRJEB62680.
