# Supplementary material for: Identification of microbial pathogens in Neolithic Scandinavian humans
Source: Sci Rep. 2024 Mar 7;14:5630. doi: 10.1038/s41598-024-56096-0 (PMC10920878; doi:10.1038/s41598-024-56096-0)
Supplement: Supplementary file 1 — Supplementary Information. [file 41598_2024_56096_MOESM1_ESM.docx]

# Identification of microbial pathogens in Neolithic Scandinavian humans

Nora Bergfeldt*^1,2,3^, Emrah Kırdök^4^, Nikolay Oskolkov^5^, Claudio Mirabello^6^, Per Unneberg^7^, Helena Malmström^8^, Magdalena Fraser^8^, Federico Sanchez-Quinto^8^, Roger Jorgensen^9^, Birgitte Skar^10^, Kerstin Lidén^11^, Mattias Jakobsson^8^, Jan Storå^11^, Anders Götherström^1,11^

*Corresponding author

1. Centre for Palaeogenetics, Stockholm University, Stockholm, Sweden

2. Department of Zoology, Stockholm University, Stockholm, Sweden

3. Department of Bioinformatics and Genetics, Swedish Museum of Natural History, Stockholm, Sweden

4. Department of Biotechnology, Faculty of Science, Mersin University, Mersin Turkiye

5. Department of Biology, National Bioinformatics Infrastructure Sweden, Science for Life Laboratory, Lund University, Lund Sweden

6. Department of Physics, Chemistry and Biology, National Bioinformatics Infrastructure Sweden, Science for Life Laboratory, Linköping University, Linköping, Sweden

7. Department of Cell and Molecular Biology, National Bioinformatics Infrastructure Sweden, Science for Life Laboratory, Uppsala University, Uppsala, Sweden

8. Human Evolution, Department of Organism Biology, Uppsala University, Uppsala, Sweden

9. Tromsø University Museum, University of Tromsø-The Arctic University of Norway, Tromsø, Norway

10. Department of Archaeology and Cultural History, NTNU University Museum, Trondheim, Norway

11. Department of Archaeology and Classical Studies, Stockholm University, Stockholm, Sweden

Supplementary information

**Table S1.** Samples including age and reference to the source of DNA extraction. * Indicates reservoir corrected calibrated dates

| **Sample** | **Location** | **Context** | **Age cal. Years BP (2 sigma)** | **Source** |
| --- | --- | --- | --- | --- |
| hum1 | Hummervikholmen | SHG | 9471–9225 * | Gunther et al. 2018 |
| hum2 | Hummervikholmen | SHG | 9732–9368 * | Gunther et al. 2018 |
| sbj001 | Stora Bjers | SHG | 8963–8579* | Gunther et al. 2018 |
| stg001 | Steigen | SHG | 5950–5764 * | Gunther et al. 2018 |
| ans004 | Ansarve | LNF | 3793-3733 | Fraser et al. 2018 |
| ans006 | Ansarve | FBC | 5040–4870 | Sanchez-Quinto et al. 2019 |
| ans007 | Ansarve | FBC | 4960–4840 | Sanchez-Quinto et al. 2019 |
| ans009 | Ansarve | FBC | 4830–4580 | Sanchez-Quinto et al. 2019 |
| ans010 | Ansarve | LNF | 3638-3578 | Fraser et al. 2018 |
| gok002 | Gökhem | FBC | 5050-4750 | Skoglund et al. 2012, 2014 |
| gok004 | Gökhem | FBC | 4971-4871 | Skoglund et al. 2012, 2014 |
| gok005 | Gökhem | FBC | 5050–4750 | Skoglund et al. 2012, 2014 |
| ros003 | Rössberga | FBC | 5280–4880 | Malmström et al. 2019 |
| ros004 | Rössberga | FBC | 5040–4870 | Malmström et al. 2019 |
| ajv58 | Ajvide | PWC | 4900–4600 | Skoglund et al. 2014; Gunther et al. 2018 |
| ajv82 | Ajvide | PWC | - | This study |
| ajv36 | Ajvide | PWC | 4961–4630 * | Coutinho et al., 2020 |
| ajv49 | Ajvide | PWC | - | This study |
| ajv54 | Ajvide | PWC | 4850–4630 | Malmström et al. 2019 |
| ajv70 | Ajvide | PWC | 4900–4600 | Skoglund et al. 2012, 2014; Gunther et al. 2018 |
| ajv59 | Ajvide | PWC | 4900–4600 * | Coutinho et al., 2020 |
| hem001 | Hemmor | PWC | 4961–4827 * | Coutinho et al., 2020 |
| hem004 | Hemmor | PWC | 5257–4862 * | Coutinho et al., 2020 |
| hem005 | Hemmor | PWC | 5289–4974 * | Coutinho et al., 2020 |
| vbj001 | Västerbjers | PWC | 4812–4423 * | Coutinho et al., 2020 |
| vbj002 | Västerbjers | PWC | 4832–4582 * | Coutinho et al., 2020 |
| vbj004 | Västerbjers | PWC | 4825–4446 * | Coutinho et al., 2020 |
| vbj006 | Västerbjers | PWC | 4870–4655 * | Coutinho et al., 2020 |
| vbj007 | Västerbjers | PWC | 4499–4247 * | Coutinho et al., 2020 |
| vbj008 | Västerbjers | PWC | 4814–4418 * | Coutinho et al., 2020 |
| vbj012 | Västerbjers | PWC | 4961–4828 * | Coutinho et al., 2020 |
| vbj013 | Västerbjers | PWC | 4833–4581 * | Coutinho et al., 2020 |
| vbj014 | Västerbjers | PWC | 4849–4576 * | Coutinho et al., 2020 |
| vbj017 | Västerbjers | PWC | 4867–4581 * | Coutinho et al., 2020 |
| vbj018 | Västerbjers | PWC | 4860–4644 * | Coutinho et al., 2020 |
| ber1 | Bergsgraven | BAC | 4570–4420 | Malmström et al. 2019 |
| ber2 | Bergsgraven | BAC | 4590–4430 | Malmström et al. 2019 |
| oll007 | Öllsjö | BAC | 4810–4450 | Malmström et al. 2019 |

**Table S2.** Species identified in the dataset

| taxID | taxName |
| --- | --- |
| 1006 | Marivirga tractuosa |
| 1007105 | Pusillimonas sp. T7-7 |
| 101571 | Burkholderia ubonensis |
| 1019 | Capnocytophaga sputigena |
| 1033854 | Thioalkalivibrio sulfidiphilus |
| 103733 | Saccharothrix syringae |
| 103855 | Bordetella hinzii |
| 104268 | Tenacibaculum mesophilum |
| 104336 | Microbacterium foliorum |
| 1049 | Allochromatium vinosum |
| 105219 | Ralstonia mannitolilytica |
| 1061 | Rhodobacter capsulatus |
| 1063 | Rhodobacter sphaeroides |
| 106590 | Cupriavidus necator |
| 106592 | Ensifer adhaerens |
| 1069 | Rhodomicrobium vannielii |
| 1076 | Rhodopseudomonas palustris |
| 1079 | Blastochloris viridis |
| 10847 | Escherichia virus phiX174 |
| 1085 | Rhodospirillum rubrum |
| 108981 | Acinetobacter schindleri |
| 1090615 | Nakamurella panacisegetis |
| 109328 | Leptotrichia trevisanii |
| 1096868 | Pseudonocardia sp. EC080625-04 |
| 11 | Cellulomonas gilvus |
| 1108 | Chloroflexus aurantiacus |
| 1108595 | Chromobacterium vaccinii |
| 110937 | Rathayibacter festucae |
| 1109743 | Streptomyces sp. SCSIO 03032 |
| 111501 | Muricauda ruestringensis |
| 114 | Gemmata obscuriglobus |
| 1155739 | Moorea producens |
| 1176649 | Agrobacterium fabrum |
| 119219 | Cupriavidus metallidurans |
| 1207504 | Burkholderia pseudomultivorans |
| 1208324 | Celeribacter indicus |
| 121719 | Pannonibacter phragmitetus |
| 122355 | Pseudomonas psychrophila |
| 1229727 | Thiobacimonas profunda |
| 123899 | Bordetella trematum |
| 1267768 | Brevirhabdus pacifica |
| 1270 | Micrococcus luteus |
| 1275 | Kocuria rosea |
| 1280 | Staphylococcus aureus |
| 1282 | Staphylococcus epidermidis |
| 1283 | Staphylococcus haemolyticus |
| 128780 | Stenotrophomonas acidaminiphila |
| 1290 | Staphylococcus hominis |
| 1291540 | Candidatus Methanomethylophilus alvus |
| 129338 | Geobacillus subterraneus |
| 1296 | Staphylococcus sciuri |
| 1297742 | Myxococcus hansupus |
| 1299 | Deinococcus radiodurans |
| 129921 | Amycolatopsis keratiniphila |
| 1302 | Streptococcus gordonii |
| 1303 | Streptococcus oralis |
| 1305 | Streptococcus sanguinis |
| 1309 | Streptococcus mutans |
| 1311 | Streptococcus agalactiae |
| 1312183 | Rhizobium jaguaris |
| 1313 | Streptococcus pneumoniae |
| 1314 | Streptococcus pyogenes |
| 131568 | Kineococcus radiotolerans |
| 132132 | Desulfomicrobium orale |
| 1328 | Streptococcus anginosus |
| 1336 | Streptococcus equi |
| 1338 | Streptococcus intermedius |
| 1351 | Enterococcus faecalis |
| 1354 | Enterococcus hirae |
| 135487 | Nocardia cyriacigeorgica |
| 1355015 | Streptomyces pluripotens |
| 1355477 | Bradyrhizobium diazoefficiens |
| 1358 | Lactococcus lactis |
| 13690 | Sphingobium yanoikuyae |
| 1379270 | Gemmatimonas phototrophica |
| 1385591 | Burkholderia sp. BDU6 |
| 1385592 | Burkholderia sp. BDU8 |
| 1397 | Bacillus circulans |
| 1398 | Bacillus coagulans |
| 1404 | Bacillus megaterium |
| 1404367 | Bradyrhizobium sp. 3(2017) |
| 1405 | Bacillus mycoides |
| 1406 | Paenibacillus polymyxa |
| 1416803 | Bordetella genomosp. 9 |
| 1420917 | Marinobacter salarius |
| 1421 | Lysinibacillus sphaericus |
| 1423 | Bacillus subtilis |
| 1427356 | Hyphomicrobium nitrativorans |
| 1428 | Bacillus thuringiensis |
| 143361 | Filifactor alocis |
| 1437360 | Bradyrhizobium erythrophlei |
| 146919 | Salinibacter ruber |
| 147645 | Paracoccus yeei |
| 1478 | Bacillus simplex |
| 147802 | Lactobacillus iners |
| 148447 | Paraburkholderia phymatum |
| 1491 | Clostridium botulinum |
| 1492 | Clostridium butyricum |
| 1493872 | Chryseobacterium shandongense |
| 1496 | Clostridioides difficile |
| 1501 | Clostridium pasteurianum |
| 1502 | Clostridium perfringens |
| 1503054 | Burkholderia stagnalis |
| 1504 | Clostridium septicum |
| 1505 | Paeniclostridium sordellii |
| 1513 | Clostridium tetani |
| 1518022 | Siphovirus contig89 |
| 152297 | Pseudoalteromonas issachenkonii |
| 152480 | Burkholderia ambifaria |
| 152682 | Sphingomonas melonis |
| 1534 | Clostridium kluyveri |
| 153496 | Kozakia baliensis |
| 1535768 | Streptomyces lunaelactis |
| 153948 | Nitrosomonas sp. AL212 |
| 154 | Spirochaeta thermophila |
| 1542 | Clostridium novyi |
| 155077 | Cellvibrio japonicus |
| 155892 | Caulobacter vibrioides |
| 1561 | Clostridium baratii |
| 1575 | Leifsonia xyli |
| 157592 | Sterolibacterium denitrificans |
| 157687 | Leptotrichia wadei |
| 157782 | Pseudomonas parafulva |
| 158 | Treponema denticola |
| 1580596 | Phaeobacter piscinae |
| 1583098 | Fusobacterium hwasookii |
| 1584 | Lactobacillus delbrueckii |
| 158836 | Enterobacter hormaechei |
| 158899 | Collimonas fungivorans |
| 160791 | Sphingomonas wittichii |
| 160808 | Acidithiobacillus ferrivorans |
| 1608628 | Candidatus Filomicrobium marinum |
| 1613 | Lactobacillus fermentum |
| 161493 | Anaeromyxobacter dehalogenans |
| 162 | Treponema phagedenis |
| 1620421 | Hoeflea sp. IMCC20628 |
| 1630135 | Dermabacter vaginalis |
| 1637869 | Burkholderia sp. MSMB0856 |
| 1639 | Listeria monocytogenes |
| 1641402 | Pseudonocardia sp. HH130629-09 |
| 164546 | Cupriavidus taiwanensis |
| 1648 | Erysipelothrix rhusiopathiae |
| 1655 | Actinomyces naeslundii |
| 1656 | Actinomyces viscosus |
| 1660 | Actinomyces odontolyticus |
| 1661 | Trueperella pyogenes |
| 1667 | Arthrobacter sp. |
| 1673076 | Sphingobium hydrophobicum |
| 167964 | Anaerolinea thermophila |
| 1680 | Bifidobacterium adolescentis |
| 1681 | Bifidobacterium bifidum |
| 1683 | Bifidobacterium angulatum |
| 168471 | Laribacter hongkongensis |
| 1690815 | Pseudonocardia sp. HH130630-07 |
| 1694 | Bifidobacterium pseudolongum |
| 169430 | Paraburkholderia hospita |
| 1697053 | Oblitimonas alkaliphila |
| 1703 | Brevibacterium linens |
| 1705310 | Burkholderia sp. IDO3 |
| 1708 | Cellulomonas fimi |
| 1710 | Cellulosimicrobium cellulans |
| 1725 | Corynebacterium xerosis |
| 173366 | Methylocystis rosea |
| 173480 | Gemmatimonas aurantiaca |
| 1735162 | Candidatus Peribacter riflensis |
| 1744 | Propionibacterium freudenreichii |
| 174633 | Candidatus Kuenenia stuttgartiensis |
| 1747 | Cutibacterium acnes |
| 1748 | Acidipropionibacterium acidipropionici |
| 1749 | Acidipropionibacterium jensenii |
| 1750 | Pseudopropionibacterium propionicum |
| 1761016 | Paraburkholderia caffeinilytica |
| 1764 | Mycobacterium avium |
| 1767 | Mycobacterium intracellulare |
| 1768 | Mycobacterium kansasii |
| 1768108 | Deinococcus actinosclerus |
| 1772 | Mycobacterium smegmatis |
| 1788 | Mycobacterium terrae |
| 1791 | Mycobacterium aurum |
| 1792 | Mycobacterium chitae |
| 1793 | Mycobacterium fallax |
| 1795630 | Frondihabitans sp. PAMC 28766 |
| 179636 | Alicycliphilus denitrificans |
| 180282 | Delftia tsuruhatensis |
| 1804 | Mycobacterium gilvum |
| 1804984 | Burkholderia sp. OLGA172 |
| 1814 | Amycolatopsis methanolica |
| 181663 | Desulfococcus oleovorans |
| 1823 | Nocardia otitidiscaviarum |
| 1824 | Nocardia asteroides |
| 182640 | Kribbella flavida |
| 1828 | Rhodococcus fascians |
| 1829 | Rhodococcus rhodochrous |
| 1830 | Rhodococcus ruber |
| 1833 | Rhodococcus erythropolis |
| 1836 | Saccharopolyspora erythraea |
| 184914 | Corallococcus coralloides |
| 1852 | Saccharomonospora viridis |
| 185642 | Mycobacterium parmense |
| 1858609 | Acidovorax sp. T1 |
| 1859 | Frankia alni |
| 1867 | Actinoplanes teichomyceticus |
| 1867719 | Phyllobacterium sp. Tri-48 |
| 1869227 | bacterium |
| 187137 | Oceanithermus profundus |
| 187493 | Thalassolituus oleivorans |
| 1877 | Micromonospora echinospora |
| 1882682 | Microvirga ossetica |
| 1884913 | Candidatus Planktophila lacus |
| 1884914 | Candidatus Planktophila dulcis |
| 1888 | Streptomyces albus |
| 1889 | Streptomyces ambofaciens |
| 1893 | Streptomyces atratus |
| 189426 | Paenibacillus odorifer |
| 1901 | Streptomyces clavuligerus |
| 1907 | Streptomyces glaucescens |
| 190721 | Ralstonia insidiosa |
| 1908 | Streptomyces globisporus |
| 1911 | Streptomyces griseus |
| 1912 | Streptomyces hygroscopicus |
| 1914 | Streptomyces lavendulae |
| 191495 | Conexibacter woesei |
| 1915 | Streptomyces lincolnensis |
| 192 | Azospirillum brasilense |
| 1927 | Streptomyces rimosus |
| 192812 | Erythrobacter flavus |
| 1931 | Streptomyces sp. |
| 1933880 | Glutamicibacter halophytocola |
| 194963 | Sinorhizobium americanum |
| 1967 | Streptomyces kanamyceticus |
| 196914 | Actinoplanes friuliensis |
| 1984801 | Streptomyces sp. CLI2509 |
| 198618 | Pseudomonas umsongensis |
| 198620 | Pseudomonas koreensis |
| 199 | Campylobacter concisus |
| 200 | Campylobacter curvus |
| 2001 | Streptosporangium roseum |
| 200451 | Pseudomonas poae |
| 2006 | Thermobispora bispora |
| 2014 | Nocardiopsis dassonvillei |
| 2014542 | Alcanivorax sp. N3-2A |
| 2026 | Thermoactinomyces vulgaris |
| 2026199 | Paraburkholderia aromaticivorans |
| 203 | Campylobacter rectus |
| 204 | Campylobacter showae |
| 2041 | Aeromicrobium erythreum |
| 2047 | Rothia dentocariosa |
| 2054 | Gordonia bronchialis |
| 2055 | Gordonia terrae |
| 2061 | Tsukamurella paurometabola |
| 2066070 | Mesorhizobium japonicum |
| 2074 | Pseudonocardia autotrophica |
| 208223 | Kosakonia cowanii |
| 210 | Helicobacter pylori |
| 216778 | Stenotrophomonas rhizophila |
| 216816 | Bifidobacterium longum |
| 217203 | Achromobacter spanius |
| 217204 | Achromobacter insolitus |
| 2173 | Methanobrevibacter smithii |
| 221822 | Phaeobacter inhibens |
| 222805 | Mycobacterium chimaera |
| 223967 | Methylobacterium populi |
| 227 | Pseudoalteromonas carrageenovora |
| 232537 | Candidatus Rhodoluna limnophila |
| 237609 | Pseudomonas alkylphenolica |
| 237610 | Pseudomonas psychrotolerans |
| 239935 | Akkermansia muciniphila |
| 240495 | Pseudonocardia dioxanivorans |
| 2434 | Roseobacter denitrificans |
| 244566 | Pseudomonas lurida |
| 246432 | Staphylococcus equorum |
| 252307 | Robiginitalea biformata |
| 253 | Chryseobacterium indologenes |
| 256618 | Parvibaculum lavamentivorans |
| 265293 | [Pseudomonas] mesoacidophila |
| 265959 | Komagataeibacter saccharivorans |
| 266 | Paracoccus denitrificans |
| 267128 | Sphingopyxis granuli |
| 2702 | Gardnerella vaginalis |
| 273384 | Brevibacterium aurantiacum |
| 274 | Thermus thermophilus |
| 2743 | Marinobacter hydrocarbonoclasticus |
| 2751 | Carnobacterium maltaromaticum |
| 279058 | Collimonas arenae |
| 279113 | Collimonas pratensis |
| 28025 | Bifidobacterium animalis |
| 28049 | Acidothermus cellulolyticus |
| 28068 | Rubrivivax gelatinosus |
| 28087 | Legionella sainthelensi |
| 28095 | Burkholderia gladioli |
| 28109 | Pseudoalteromonas nigrifaciens |
| 28112 | Tannerella forsythia |
| 28116 | Bacteroides ovatus |
| 28131 | Prevotella intermedia |
| 28132 | Prevotella melaninogenica |
| 28197 | Arcobacter butzleri |
| 28198 | Arcobacter cryaerophilus |
| 283811 | Stackebrandtia nassauensis |
| 28447 | Clavibacter michiganensis |
| 28448 | Komagataeibacter xylinus |
| 28450 | Burkholderia pseudomallei |
| 285 | Comamonas testosteroni |
| 287 | Pseudomonas aeruginosa |
| 288000 | Bradyrhizobium sp. BTAi1 |
| 28901 | Salmonella enterica |
| 292 | Burkholderia cepacia |
| 292800 | Flavonifractor plautii |
| 293 | Brevundimonas diminuta |
| 29385 | Staphylococcus saprophyticus |
| 29391 | Gemella morbillorum |
| 294 | Pseudomonas fluorescens |
| 29447 | Xanthomonas albilineans |
| 29449 | Rhizobium etli |
| 29466 | Veillonella parvula |
| 29542 | Pelobacter acetylenicus |
| 29549 | Rhodothermus marinus |
| 29570 | Halomonas meridiana |
| 296 | Pseudomonas fragi |
| 296587 | Micromonas commoda |
| 300 | Pseudomonas mendocina |
| 301 | Pseudomonas oleovorans |
| 303 | Pseudomonas putida |
| 304895 | Catenulispora acidiphila |
| 305 | Ralstonia solanacearum |
| 3055 | Chlamydomonas reinhardtii |
| 3075 | Auxenochlorella protothecoides |
| 312306 | Pseudomonas entomophila |
| 313588 | Croceibacter atlanticus |
| 313603 | Maribacter sp. HTCC2170 |
| 314722 | Pseudoxanthomonas suwonensis |
| 315405 | Streptococcus gallolyticus |
| 316 | Pseudomonas stutzeri |
| 317 | Pseudomonas syringae |
| 31958 | Amycolatopsis orientalis |
| 32002 | Achromobacter denitrificans |
| 321846 | Pseudomonas simiae |
| 329 | Ralstonia pickettii |
| 33010 | Cutibacterium avidum |
| 330214 | Nitrospira defluvii |
| 33033 | Parvimonas micra |
| 33050 | Sphingopyxis macrogoltabida |
| 33059 | Acidithiobacillus caldus |
| 330922 | Psychrobacter cryohalolentis |
| 332163 | Candidatus Solibacter usitatus |
| 334542 | Rhodococcus qingshengii |
| 337 | Burkholderia glumae |
| 339 | Xanthomonas campestris |
| 33903 | Streptomyces avermitilis |
| 33910 | Amycolatopsis mediterranei |
| 34 | Myxococcus xanthus |
| 34018 | Rhodospirillum centenum |
| 34029 | Leptothrix cholodnii |
| 34062 | Moraxella osloensis |
| 34073 | Variovorax paradoxus |
| 34085 | Riemerella anatipestifer |
| 34105 | Streptobacillus moniliformis |
| 342113 | Burkholderia oklahomensis |
| 343 | Xanthomonas translucens |
| 346 | Xanthomonas citri |
| 347 | Xanthomonas oryzae |
| 348824 | Rhizobium favelukesii |
| 35 | Myxococcus macrosporus |
| 353 | Azotobacter chroococcum |
| 354 | Azotobacter vinelandii |
| 35554 | Geobacter sulfurreducens |
| 357276 | Bacteroides dorei |
| 35762 | Planobispora rosea |
| 358 | Agrobacterium tumefaciens |
| 35806 | Rhodovulum sulfidophilum |
| 35814 | Bordetella holmesii |
| 358220 | Acidovorax sp. KKS102 |
| 364029 | Comamonas sp. E6 |
| 364317 | Verminephrobacter eiseniae |
| 364410 | Granulibacter bethesdensis |
| 36809 | Mycobacterium abscessus |
| 36861 | Thiobacillus denitrificans |
| 37326 | Nocardia brasiliensis |
| 37329 | Nocardia farcinica |
| 37330 | Nocardia nova |
| 37332 | Nocardia seriolae |
| 374606 | Aminobacter sp. MSH1 |
| 374840 | Enterobacteria phage phiX174 sensu lato |
| 375 | Bradyrhizobium japonicum |
| 376 | Bradyrhizobium sp. |
| 379067 | Streptomyces bingchenggensis |
| 37919 | Rhodococcus opacus |
| 379347 | Ruegeria mobilis |
| 380 | Sinorhizobium fredii |
| 380021 | Pseudomonas protegens |
| 381 | Mesorhizobium loti |
| 382 | Sinorhizobium meliloti |
| 38289 | Corynebacterium jeikeium |
| 38300 | Streptomyces pristinaespiralis |
| 384 | Rhizobium leguminosarum |
| 388357 | Kocuria turfanensis |
| 39478 | Streptomyces laurentii |
| 396 | Rhizobium phaseoli |
| 39645 | Mesorhizobium ciceri |
| 399 | Neorhizobium galegae |
| 40137 | Oligotropha carboxidovorans |
| 40214 | Acinetobacter johnsonii |
| 40216 | Acinetobacter radioresistens |
| 40269 | Aliivibrio salmonicida |
| 40318 | Streptomyces nodosus |
| 40324 | Stenotrophomonas maltophilia |
| 40559 | Botrytis cinerea |
| 408 | Methylobacterium extorquens |
| 41 | Stigmatella aurantiaca |
| 412690 | Microterricola viridarii |
| 412963 | Paraburkholderia rhizoxinica |
| 41899 | Burkholderia plantarii |
| 41977 | Azoarcus communis |
| 42197 | Actinosynnema pretiosum |
| 42253 | Nitrospira moscoviensis |
| 42256 | Rubrobacter radiotolerans |
| 43348 | Mycobacterium branderi |
| 434010 | Hydrogenophaga sp. PBL-H3 |
| 435897 | Lysobacter capsici |
| 43675 | Rothia mucilaginosa |
| 43767 | Rhodococcus hoagii |
| 43768 | Corynebacterium matruchotii |
| 43771 | Corynebacterium urealyticum |
| 44008 | Enterococcus cecorum |
| 44056 | Aureococcus anophagefferens |
| 442 | Gluconobacter oxydans |
| 445709 | Pandoraea thiooxydans |
| 44577 | Nitrosomonas ureae |
| 446 | Legionella pneumophila |
| 450378 | Croceicoccus marinus |
| 45243 | Capnocytophaga haemolytica |
| 45398 | Streptomyces griseoviridis |
| 455432 | Nocardia terpenica |
| 45617 | Human endogenous retrovirus K |
| 45634 | Streptococcus cristatus |
| 458033 | Chloracidobacterium thermophilum |
| 459526 | Flavobacterium anhuiense |
| 45972 | Staphylococcus pasteuri |
| 463025 | Bordetella bronchialis |
| 466153 | Singulisphaera acidiphila |
| 46679 | Pseudomonas mucidolens |
| 470 | Acinetobacter baumannii |
| 470934 | Pantoea vagans |
| 471827 | Candidatus Desulforudis audaxviator |
| 47421 | Hydrogenophaga pseudoflava |
| 47671 | Lautropia mirabilis |
| 47763 | Streptomyces lydicus |
| 477641 | Modestobacter marinus |
| 47850 | Micromonospora aurantiaca |
| 47878 | Pseudomonas azotoformans |
| 47879 | Pseudomonas corrugata |
| 47880 | Pseudomonas fulva |
| 47883 | Pseudomonas synxantha |
| 47884 | Pseudomonas taetrolens |
| 47885 | Pseudomonas oryzihabitans |
| 47917 | Serratia fonticola |
| 482957 | Burkholderia lata |
| 48466 | Prosthecobacter vanneervenii |
| 485 | Neisseria gonorrhoeae |
| 486 | Neisseria lactamica |
| 48664 | Xanthomonas fragariae |
| 486698 | Mycobacterium riyadhense |
| 487 | Neisseria meningitidis |
| 4876 | Rhizophagus intraradices |
| 488 | Neisseria mucosa |
| 488447 | Burkholderia contaminans |
| 488731 | Burkholderia seminalis |
| 488732 | Burkholderia diffusa |
| 49319 | Rubrobacter xylanophilus |
| 495 | Neisseria elongata |
| 4952 | Yarrowia lipolytica |
| 496014 | Rubrobacterineae bacterium BR7-21 |
| 497727 | Candidatus Nitrososphaera gargensis |
| 5059 | Aspergillus flavus |
| 511 | Alcaligenes faecalis |
| 51123 | [Eubacterium] saphenum |
| 5127 | Fusarium fujikuroi |
| 515393 | Pseudomonas yamanorum |
| 518 | Bordetella bronchiseptica |
| 521 | Bordetella avium |
| 52584 | Brachyspira pilosicoli |
| 52773 | Actinomyces meyeri |
| 529 | Ochrobactrum anthropi |
| 531813 | Pelagibacterium halotolerans |
| 53358 | Intrasporangium calvum |
| 53399 | Hyphomicrobium denitrificans |
| 53408 | Pseudomonas citronellolis |
| 53409 | Pseudomonas coronafaciens |
| 536 | Chromobacterium violaceum |
| 53635 | Acidimicrobium ferrooxidans |
| 536441 | Chryseobacterium taklimakanense |
| 539 | Eikenella corrodens |
| 544580 | Actinomyces oris |
| 54571 | Streptomyces venezuelae |
| 546160 | Dietzia lutea |
| 548 | Klebsiella aerogenes |
| 549 | Pantoea agglomerans |
| 550 | Enterobacter cloacae |
| 5507 | Fusarium oxysporum |
| 553 | Pantoea ananatis |
| 553814 | Acidovorax sp. NA3 |
| 55518 | Magnetospirillum gryphiswaldense |
| 55601 | Vibrio anguillarum |
| 56 | Sorangium cellulosum |
| 562 | Escherichia coli |
| 56448 | Xanthomonas arboricola |
| 56459 | Xanthomonas vasicola |
| 56460 | Xanthomonas vesicatoria |
| 56646 | Fusarium venenatum |
| 56730 | Rhizobium gallicum |
| 570505 | Methylobacterium bullatum |
| 571913 | Luteipulveratus mongoliensis |
| 573 | Klebsiella pneumoniae |
| 575 | Raoultella planticola |
| 5755 | Acanthamoeba castellanii |
| 576610 | Polynucleobacter necessarius |
| 576611 | Polynucleobacter asymbioticus |
| 57704 | Tsukamurella tyrosinosolvens |
| 57706 | Citrobacter braakii |
| 5778 | Vermamoeba vermiformis |
| 57975 | Burkholderia thailandensis |
| 582 | Morganella morganii |
| 584 | Proteus mirabilis |
| 584609 | Tenacibaculum jejuense |
| 585 | Proteus vulgaris |
| 5866 | Babesia bigemina |
| 587753 | Pseudomonas chlororaphis |
| 588596 | Rhizophagus irregularis |
| 588932 | Brevundimonas naejangsanensis |
| 60550 | Burkholderia pyrrocinia |
| 60552 | Burkholderia vietnamiensis |
| 60890 | Phaeobacter gallaeciensis |
| 60920 | Sanguibacter keddieii |
| 615 | Serratia marcescens |
| 61624 | Paenibacillus mucilaginosus |
| 61647 | Pluralibacter gergoviae |
| 61648 | Kluyvera intermedia |
| 62322 | Shewanella baltica |
| 630 | Yersinia enterocolitica |
| 63186 | Zobellia galactanivorans |
| 632 | Yersinia pestis |
| 633 | Yersinia pseudotuberculosis |
| 640511 | Burkholderia sp. CCGE1002 |
| 64104 | Bacillus pseudomycoides |
| 644 | Aeromonas hydrophila |
| 645 | Aeromonas salmonicida |
| 649831 | Actinoplanes sp. N902-109 |
| 651 | Aeromonas media |
| 652 | Aeromonas schubertii |
| 654 | Aeromonas veronii |
| 65555 | Desulfocapsa sulfexigens |
| 65741 | Pseudomonas knackmussii |
| 666 | Vibrio cholerae |
| 666685 | Rhodanobacter denitrificans |
| 671143 | Candidatus Methylomirabilis oxyfera |
| 67267 | Streptomyces alboflavus |
| 67304 | Streptomyces griseorubiginosus |
| 673372 | Vibrio casei |
| 673862 | Candidatus Babela massiliensis |
| 68203 | Streptomyces fungicidicus |
| 68214 | Streptomyces griseochromogenes |
| 68270 | Streptomyces spectabilis |
| 68570 | Streptomyces albulus |
| 68895 | Cupriavidus basilensis |
| 69 | Lysobacter enzymogenes |
| 69218 | Enterobacter cancerogenus |
| 69966 | Macrococcus caseolyticus |
| 701042 | Mycobacterium marseillense |
| 702115 | Pseudomonas arsenicoxydans |
| 703222 | Kibdelosporangium sp. MJ126-NF4 |
| 712270 | Dietzia sp. oral taxon 368 |
| 712710 | Tannerella sp. oral taxon HOT-286 |
| 714 | Aggregatibacter actinomycetemcomitans |
| 717785 | Hyphomicrobium sp. MC1 |
| 722731 | Mycobacterium shigaense |
| 727 | Haemophilus influenzae |
| 729 | Haemophilus parainfluenzae |
| 731 | Histophilus somni |
| 732 | Aggregatibacter aphrophilus |
| 74426 | Collinsella aerofaciens |
| 74829 | Pseudomonas balearica |
| 75105 | Paraburkholderia caribensis |
| 754476 | Methylophaga nitratireducenticrescens |
| 75612 | Pseudomonas mandelii |
| 756892 | Acinetobacter indicus |
| 75697 | Castellaniella defragrans |
| 758782 | Burkholderia sp. THE68 |
| 758793 | Burkholderia sp. RPE64 |
| 758796 | Burkholderia sp. RPE67 |
| 758826 | Acidovorax radicis |
| 76021 | Amycolatopsis coloradensis |
| 76124 | [Eubacterium] minutum |
| 76594 | Cellulophaga baltica |
| 76758 | Pseudomonas orientalis |
| 76759 | Pseudomonas monteilii |
| 76761 | Pseudomonas veronii |
| 76775 | Malassezia restricta |
| 76853 | Solibacillus silvestris |
| 78331 | Mycobacterium canettii |
| 78587 | Asticcacaulis excentricus |
| 79329 | Chitinophaga pinensis |
| 80816 | Haliangium ochraceum |
| 80842 | Herbaspirillum rubrisubalbicans |
| 80866 | Delftia acidovorans |
| 80869 | Acidovorax citrulli |
| 81028 | Treponema brennaborense |
| 81462 | Thermanaerovibrio acidaminovorans |
| 817 | Bacteroides fragilis |
| 821 | Bacteroides vulgatus |
| 824 | Campylobacter gracilis |
| 82633 | Cupriavidus pauculus |
| 82654 | Pseudanabaena sp. PCC 7367 |
| 82996 | Serratia plymuthica |
| 83262 | Mycobacterium immunogenum |
| 83291 | Streptomyces armeniacus |
| 83564 | Desulfosarcina ovata |
| 83656 | Streptomyces tsukubensis |
| 837 | Porphyromonas gingivalis |
| 83791 | Ulva flexuosa |
| 84110 | Slackia heliotrinireducens |
| 84112 | Eggerthella lenta |
| 84531 | Lysobacter antibioticus |
| 851 | Fusobacterium nucleatum |
| 853 | Faecalibacterium prausnitzii |
| 85698 | Achromobacter xylosoxidans |
| 859 | Fusobacterium necrophorum |
| 861299 | Gemmatirosa kalamazoonesis |
| 86265 | Pseudomonas thivervalensis |
| 86664 | Bacillus flexus |
| 87883 | Burkholderia multivorans |
| 879274 | Shinella sp. HZN7 |
| 881 | Desulfovibrio vulgaris |
| 881260 | Enterobacter bugandensis |
| 88688 | Caulobacter segnis |
| 897 | Desulfococcus multivorans |
| 9 | Buchnera aphidicola |
| 92945 | Ketogulonicigenium vulgare |
| 930166 | Pseudomonas brassicacearum |
| 931 | Thiobacillus thioparus |
| 93218 | Pandoraea apista |
| 93220 | Pandoraea pnomenusa |
| 93222 | Pandoraea sputorum |
| 93944 | Nonomuraea gerenzanensis |
| 94132 | Ramlibacter tataouinensis |
| 94624 | Bordetella petrii |
| 946333 | Rhizobacter gummiphilus |
| 947919 | Desulfosarcina widdelii |
| 95485 | Burkholderia stabilis |
| 95486 | Burkholderia cenocepacia |
| 960 | Bacteriovorax stolpii |
| 9606 | Homo sapiens |
| 96344 | Cupriavidus oxalaticus |
| 96345 | Flavobacterium psychrophilum |
| 964 | Herbaspirillum seropedicae |
| 979 | Cellulophaga lytica |
| 980427 | Deinococcus wulumuqiensis |
| 985 | Cytophaga hutchinsonii |
| 986 | Flavobacterium johnsoniae |
| 996 | Flavobacterium columnare |
| 996801 | Polaribacter reichenbachii |
| 99802 | Spirometra erinaceieuropaei |


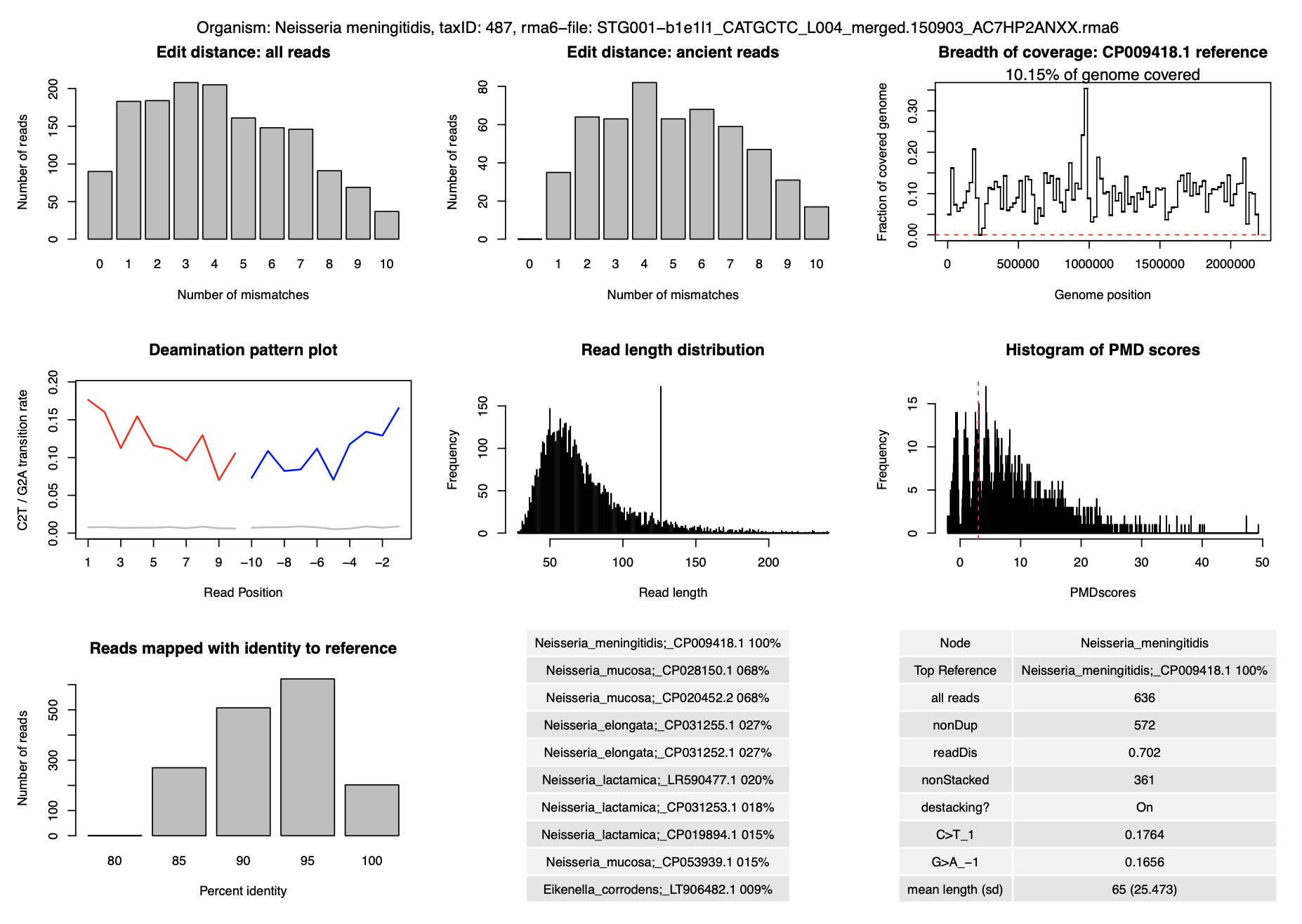


**Figure S1.** Edit distance, breadth of coverage, deamination plot, read length distribution of *Neisseria meningitidis* in individual stg001


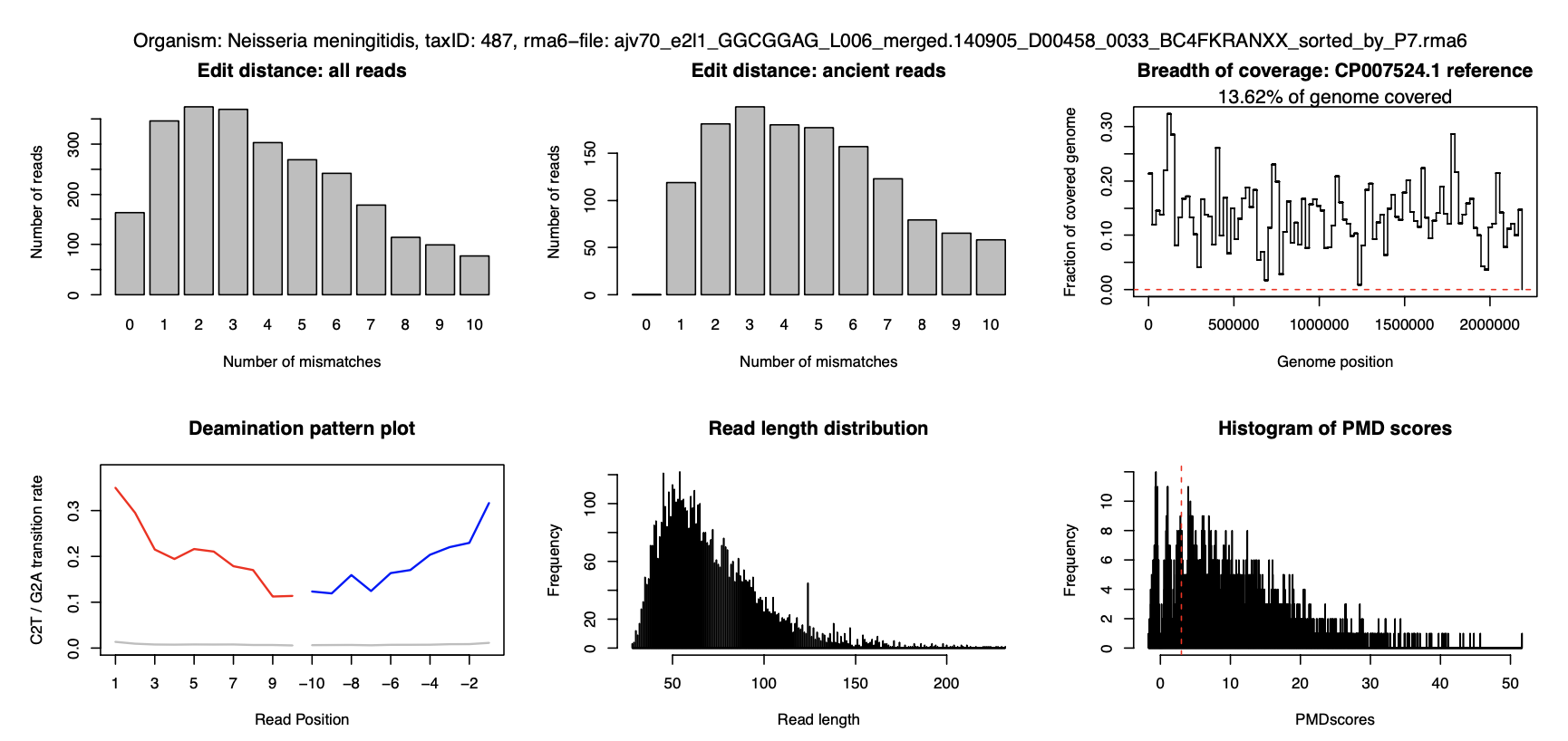


**Figure S2.** Edit distance, breadth of coverage, deamination plot, read length distribution of *Neisseria meningitidis* in individual ajv70


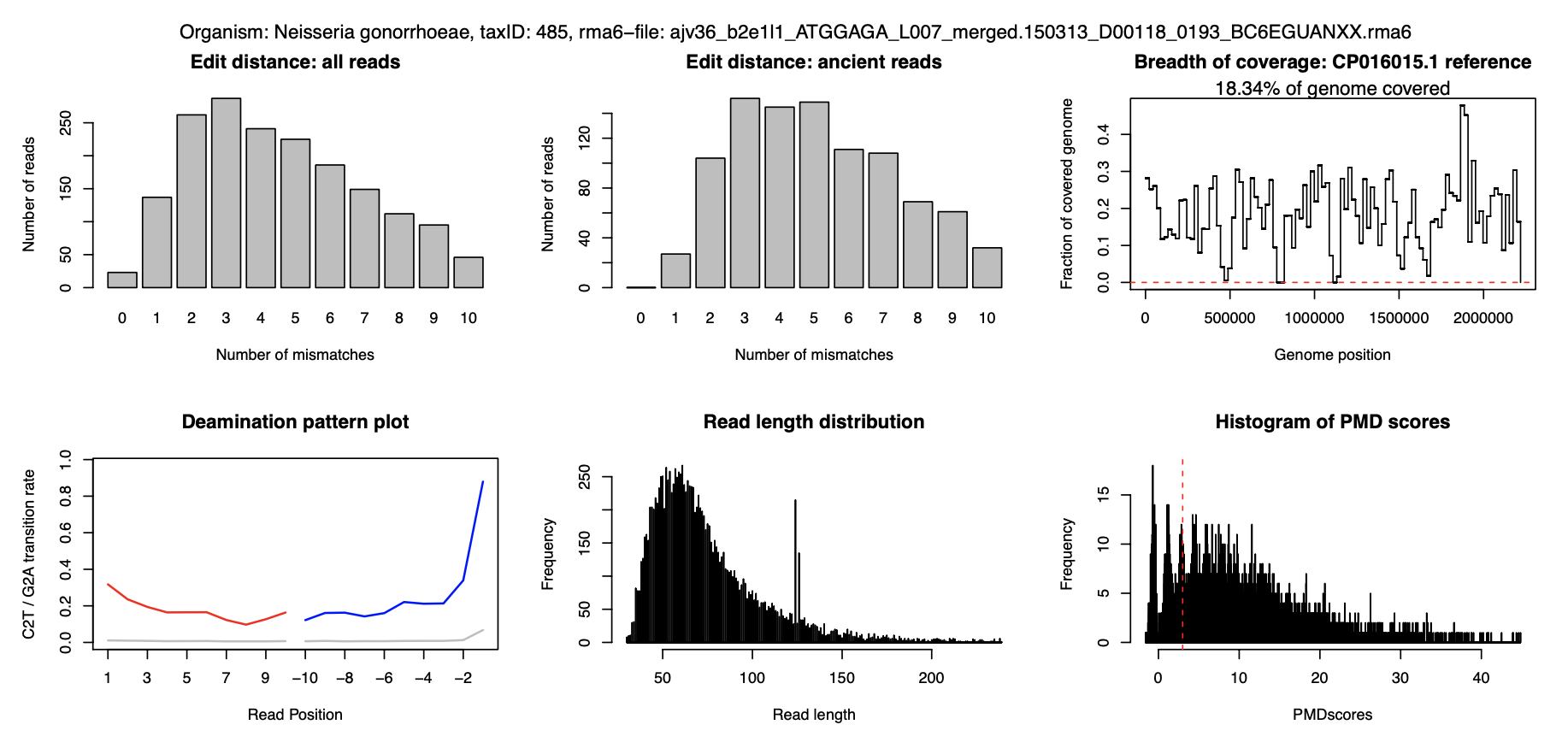


**Figure S3.** Edit distance, breadth of coverage, deamination plot, read length distribution of *Neisseria gonorrhoeae* in individual ajv36


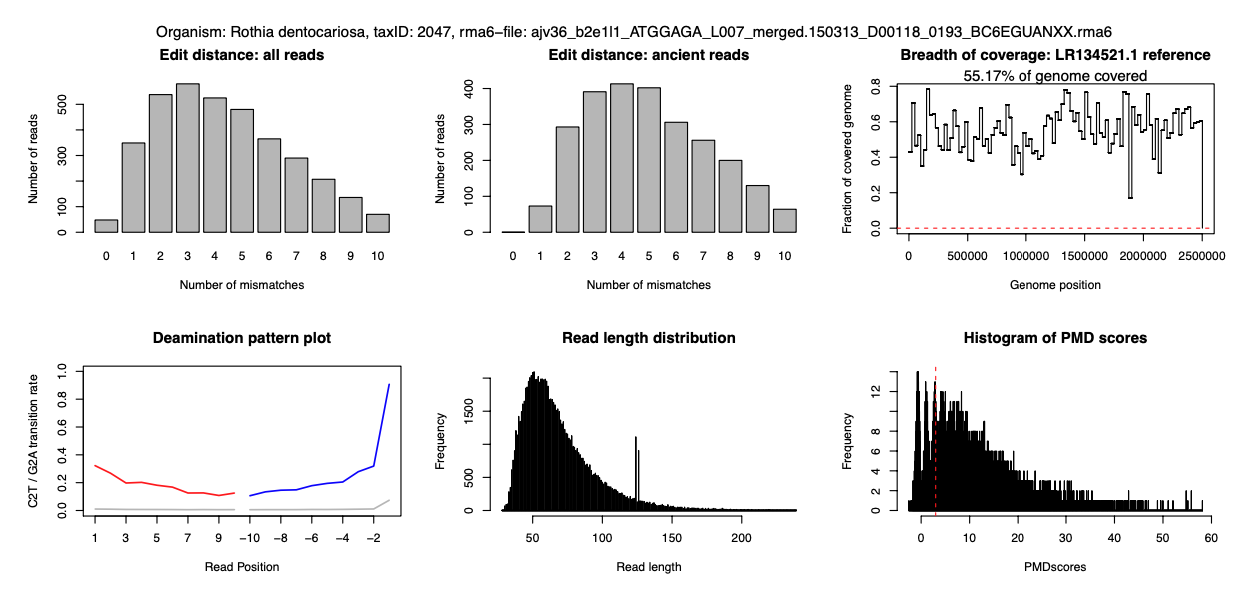


**Figure S4.** Edit distance, breadth of coverage, deamination plot, read length distribution of *Rothia dentocariosa* in individual ajv36


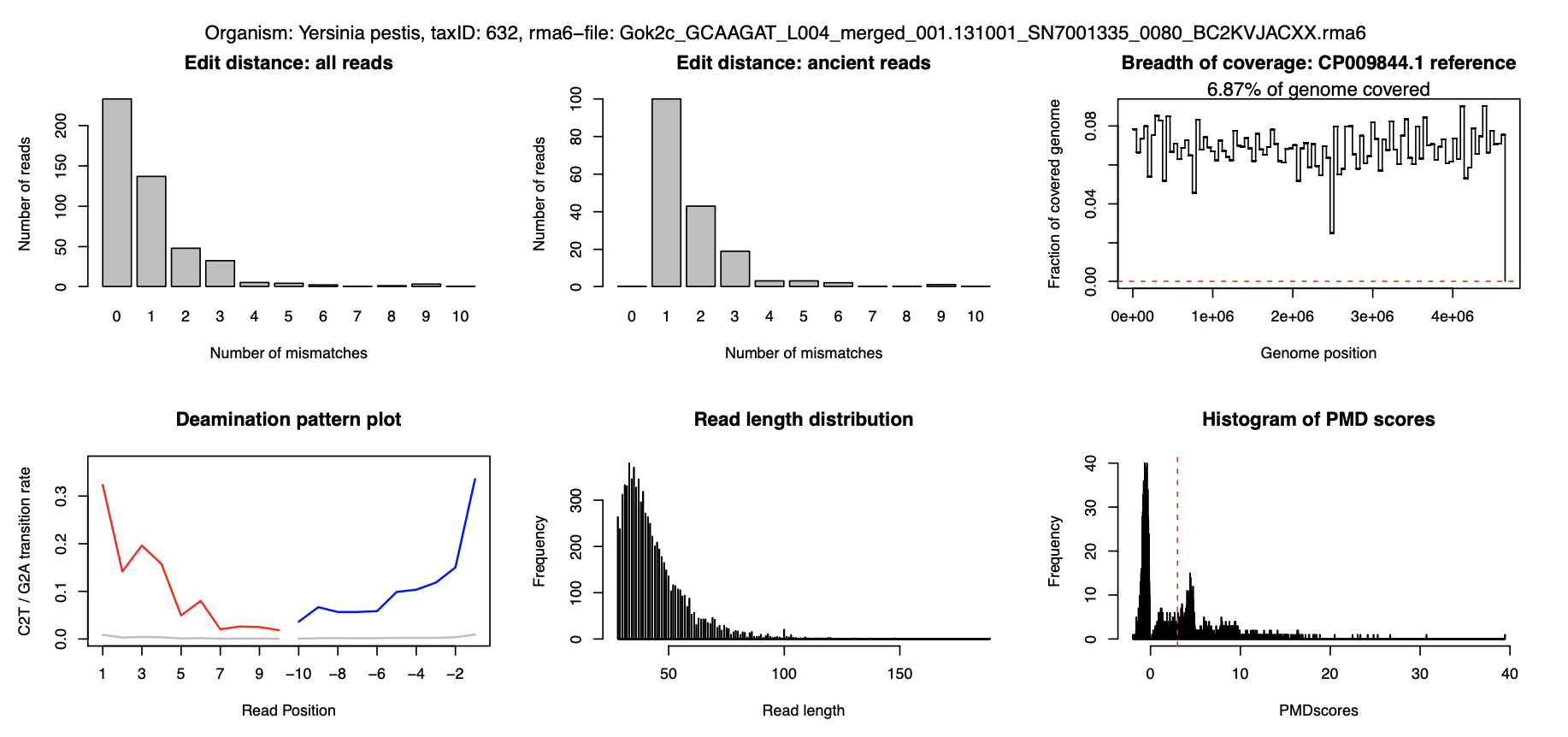


**Figure S5.** Edit distance, breadth of coverage, deamination plot, read length distribution of *Yersinia pestis* in individual gok2


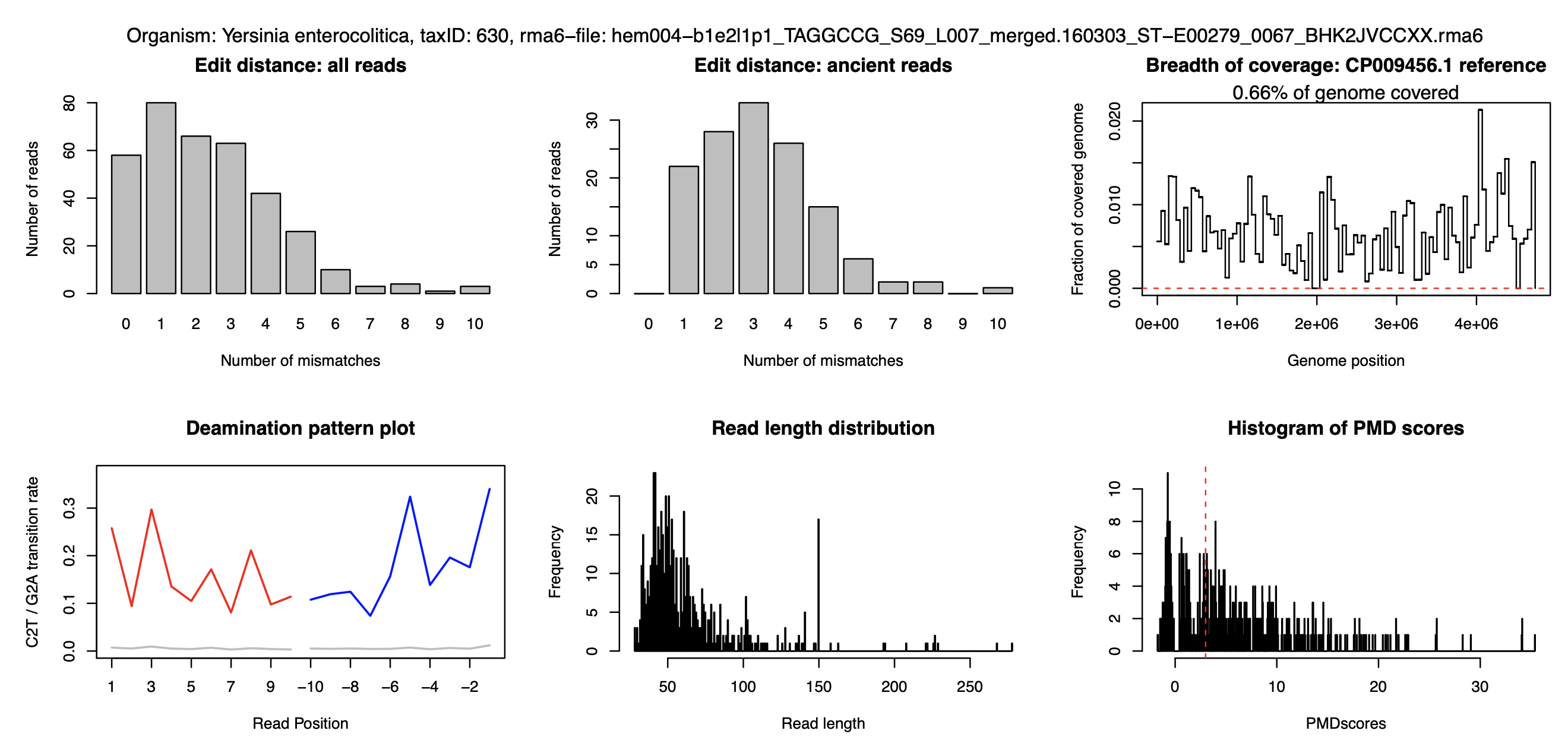


**Figure S6.** Edit distance, breadth of coverage, deamination plot, read length distribution of *Yersinia enterocolitica* in individual hem004


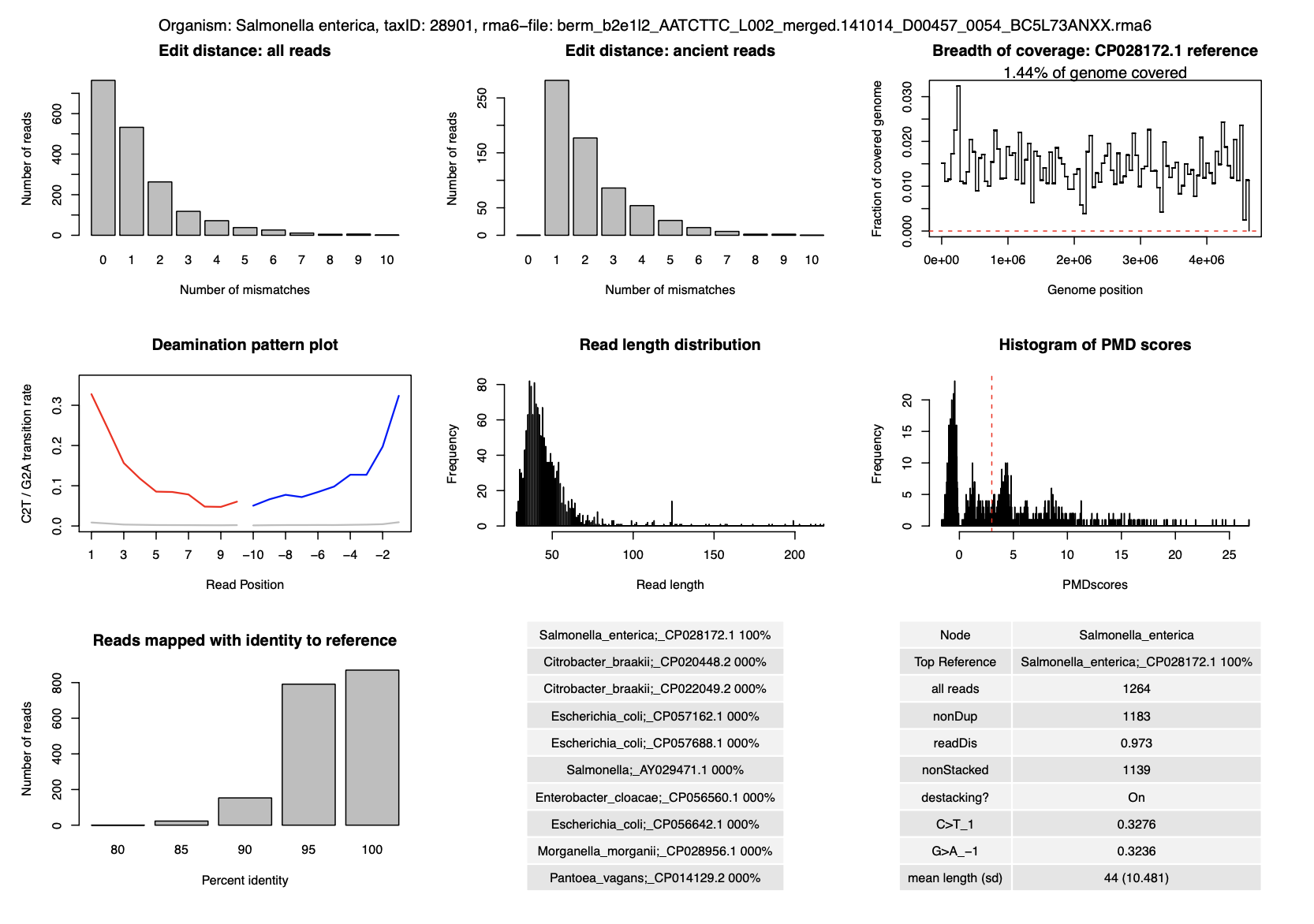


**Figure S7.** Edit distance, breadth of coverage, deamination plot, read length distribution of *Salmonella enterica* in individual ber1


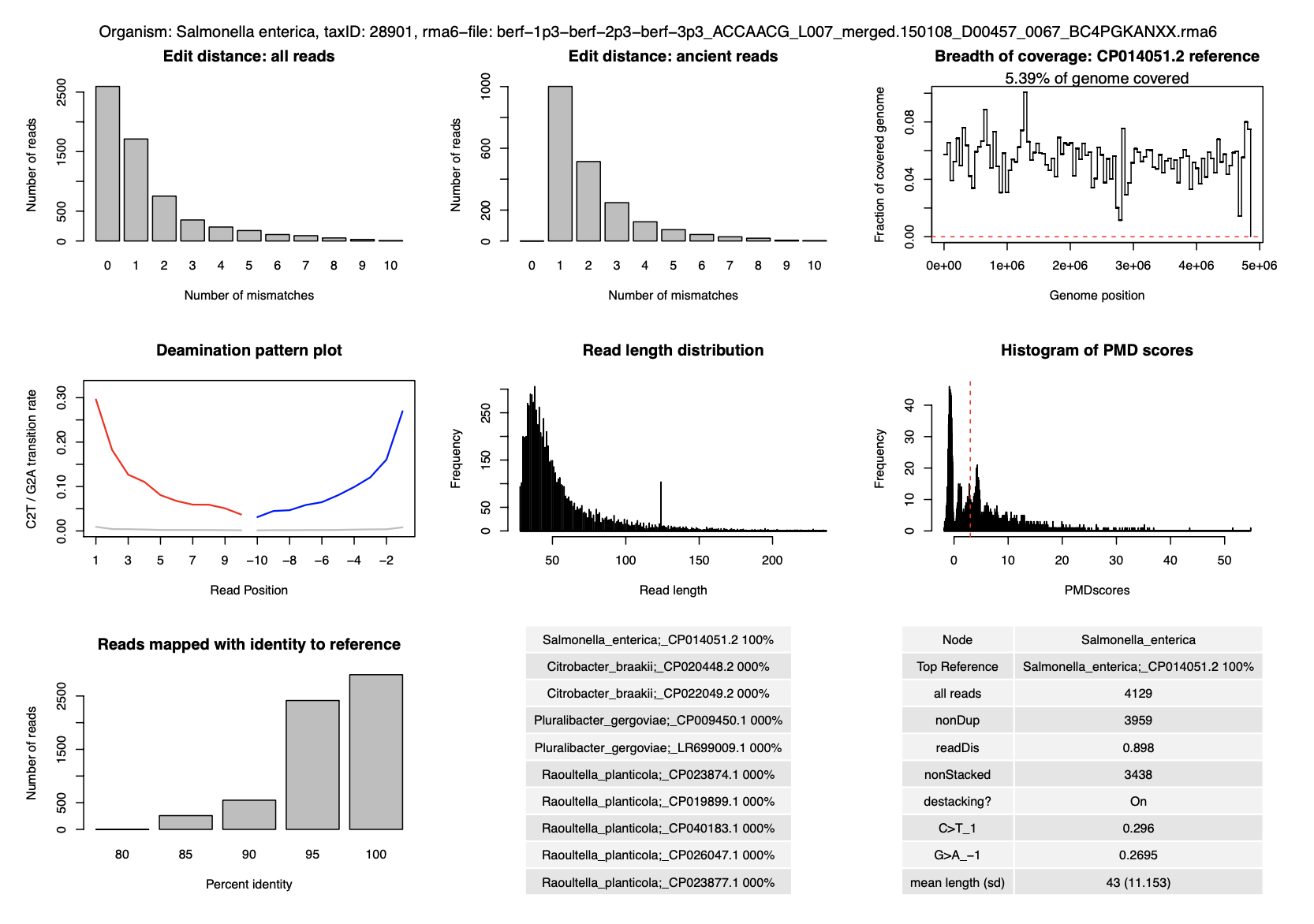


**Figure S8.** Edit distance, breadth of coverage, deamination plot, read length distribution of *Salmonella enterica* in individual Ber2


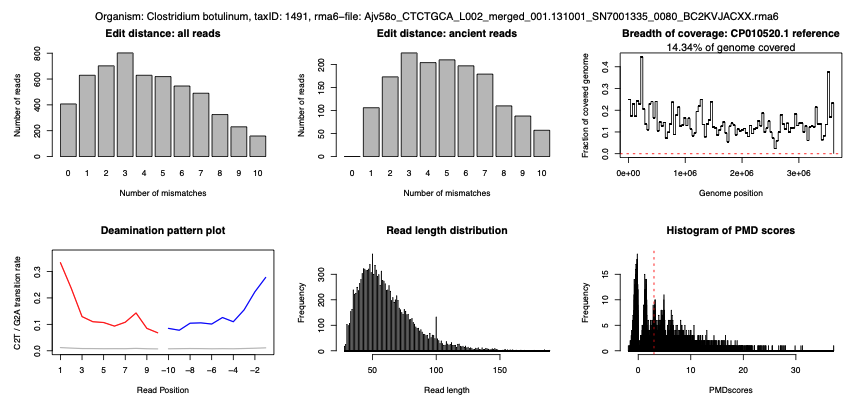


**Figure S9.** Edit distance, breadth of coverage, deamination plot, read length distribution of *Clostridium botulinum* in individual ajv58


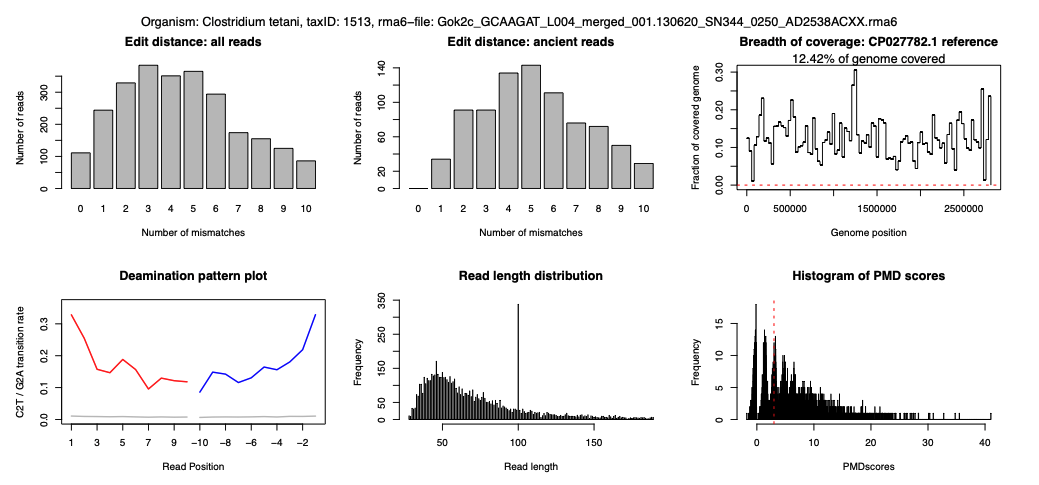


**Figure S10.** Edit distance, breadth of coverage, deamination plot, read length distribution of *Clostridium tetani* in individual gok2
